# Epidemiological‐clinical and paraclinical particularities of acute coronary syndrome without persistent ST‐segment elevation in type 2 diabetes mellitus: Retrospective comparative study in a Malagasy population

**DOI:** 10.1002/edm2.383

**Published:** 2022-10-17

**Authors:** Sitraka Angelo Raharinavalona, Rija Eric Raherison, Thierry Razanamparany, Tsikinirina Valisoa Randrianomanana, Andrianirina Dave Patrick Rakotomalala

**Affiliations:** ^1^ Internal Medicine and Cardiovascular Diseases departments Soavinandriana Hospital Center Antananarivo Madagascar; ^2^ Endocrinology Department Joseph Raseta Befelatanana University Hospital Center Antananarivo Madagascar

**Keywords:** acute coronary syndrome, cardiovascular risk factors, non‐ST‐segment elevation acute coronary syndrome, type 2 diabetes mellitus

## Abstract

**Introduction:**

This study aimed at determining the epidemiological‐clinical and paraclinical particularities of non‐ST‐segment elevation acute coronary syndrome (NSTE‐ACS) in Malagasy with type 2 diabetes mellitus (T2DM).

**Methods:**

This was a retrospective, descriptive and comparative study between patients with and without T2DM, carried out over a period of 38 months. The diagnosis of NSTE‐ACS was retained in front of the association of chest discomfort, electrical abnormalities and elevations beyond fivefold the upper reference limit of high‐sensitivity cardiac troponin.

**Result:**

With 130 patients included, the overall prevalence of NSTE‐ACS was 4.1%, of which 68 patients (52.3%) had T2DM. Compared to without T2DM, NSTE‐ACS in T2DM was characterized by young age (*p* = .0002), high‐frequency hypertension (OR 2.92 [1.23–7.25]; *p* = .0041), overweight/obesity (OR 4.39 [1.72–12.4]; *p* = .0002) and microalbuminuria (*p* < .0001), accelerated heart rate (*p* = .0104), atypical chest discomfort (OR 5.57 [2.21–15.7]; *p* < .0001), pulmonary crepitations (OR 2.25 [1.02–5.14]; *p* = .0224), high GRACE score (*p* = .0016), damage of extensive anterior leads (OR 2.11 [1.02–4.98]; *p* = .0402) and septal lead (OR 3.64 [1.41–10.3]; *p* = .0015), significant increase in cardiac troponin (*p* < .0001), high left ventricular filling pressure (OR 3.39 [1.51–7.90]; *p* = .001).

**Conclusion:**

NSTE‐ACS in T2DM is frequent, with an atypical clinical and severe paraclinical presentations. Adequate and multidisciplinary management of cardiovascular risk factors, including T2DM, could thus minimize the occurrence of NSTE‐ACS and improve this profile.

## Graphical abstract

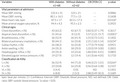

## 1 BACKGROUND

Acute coronary syndrome without ST‐segment elevation or non‐ST‐segment elevation acute coronary syndrome (NSTE‐ACS) is a source of heavy morbidity and mortality. In developed countries, its prevalence had increased by 16.7% in the space of 20 years. However, the mortality rate had decreased with the increased use of early angiography and percutaneous coronary revascularization.^1^ In Sub‐Saharan Africa, the incidence of acute coronary syndrome (ACS) is also on the rise due to more efficient diagnostic means and changes in lifestyle in urban areas. This incidence had increased from 4.1% in 1990 to 13.5% in 2013. The intrahospital mortality rate is also high, ranging from 6 to 10%.^2^, ^3^, ^4^

In addition, diabetes mellitus is also a real public health problem. The exponential increase in its frequency is mainly observed in Sub‐Saharan Africa, around 134%, according to the International Diabetes Federation.^5^ Cardiovascular and especially coronary diseases represent the leading cause of morbidity and mortality in diabetics.^6^ Indeed, diabetes entails a relative risk of atherosclerosis of 2–4 for coronary insufficiency.^7^ In addition, diabetic coronary artery disease has the particularity of being often multivessel and painless due to the associated sensory neuropathy. This justifies systematic screening for silent ischaemia in high‐risk diabetics.^8^

Particularly, in Madagascar, we have only very few references dealing with NSTE‐ACS and type 2 diabetes mellitus (T2DM) at the same time. Thus, we propose this study with the aim of determining the epidemiological‐clinical and paraclinical particularities of NSTE‐ACS in a Malagasy population with T2DM.

## 2 MATERIALS AND METHODS

### 2.1 Study design and patients

The study was carried out in the Internal Medicine and Cardiovascular Diseases departments of the Soavinandrina Hospital Center (Military Hospital), located in the 3rd district of Antananarivo, the capital of Madagascar. This was a descriptive and comparative retrospective study, conducted over a period of 38 months from November 2018 to December 2021.

The study population (N) consists of the two groups of patients with NSTE‐ACS of which the 1st group were with T2DM (n1) and the 2nd group without (n2).

The diagnosis of NSTE‐ACS was retained in front of the association of typical or atypical chest discomfort and/or other atypical signs; an electrocardiogram (ECG) showing transient ST‐segment elevation, persistent or transient ST‐segment depression, T‐wave inversion, flat T waves, or pseudo normalization of T waves; and elevations beyond fivefold the upper reference limit of high‐sensitivity cardiac troponin.^1^ The diagnosis of T2DM was made in accordance with the criteria of the American Diabetes Association (ADA).^8^

We excluded from this study incomplete records and cardiac and non‐cardiac conditions can mimic a NSTE‐ACS in front of chest discomfort.^1^

### 2.2 Clinical and laboratory data

The parameters studied were demographic data (gender and age); associated cardiovascular risk factors (hypertension, smoking, dyslipidemia, microalbuminuria and overweight/obesity); T2DM (duration, glycated haemoglobin and associated degenerative complications); and NSTE‐ACS (blood pressure, heart rate and arterial oxygen saturation on admission, clinical manifestations, ECG signs, cardiac troponin, echocardiographic signs, GRACE [Global Registry of Acute Coronary Events] score, drug treatment received during hospitalization and intra‐hospital evolution).

The enzymatic method was used for the determinations of glycated haemoglobin and serum lipids. Diabetes was said to be balanced if the Hb A1c was less than 7% (<53 mmol/mol). The presence of diabetic nephropathy is attested in the presence of pathological albuminuria, with or without alteration of the glomerular filtration rate.^9^ The diagnosis of retinopathy is made in the presence of abnormalities on examination of the vitreous and the fundus after pupillary dilation. Peripheral diabetic neuropathy is suggested by the presence of symmetrical distal sensory symptoms beginning in the lower limbs and/or impaired foot sensitivity on examination with a 10 g monofilament.^10^ The existence of arteriopathy of the lower limbs or supra‐aortic trunk was confirmed by arterial echodoppler. Hypertension was defined as systolic/diastolic blood pressure ≥ 140/90 mmHg. The cardiovascular risk was estimated according to the criteria of the European Society of Cardiology (ESC) 2019. Low‐density lipoprotein cholesterol (LDL‐C) must be <1.4 mmol/L (<55 mg/dL) in patient very high risk; <1.8 mmol/L (<70 mg/dL) if at high risk; <2.6 mmol/L (<100 mg/dL) if at moderate risk and <3.0 mmol/L (<116 mg/dL) if at low risk.^11^ Patients with LDL‐C outside these targets or taking a lipid‐lowering drug were considered to have dyslipidemia.

According to the Killip classification, we distinguish the class I: with no clinical signs of heart failure; II: with rales in the lungs, third heart sound (S3), and elevated jugular venous pressure; III: with acute pulmonary oedema; and IV: with cardiogenic shock or arterial hypotension (measured as systolic blood pressure < 90 mmHg), and evidence of peripheral vasoconstriction (oliguria, cyanosis, and diaphoresis).^12^

Typical chest discomfort is characterized by a retrosternal sensation of pain, pressure, or heaviness (‘angina’) radiating to the left arm, both arms, the right arm, the neck, or the jaw, which may be intermittent (usually lasting several minutes) or persistent. Cardiac troponin assay was made by chemiluminescence technique on Alinity Abbott 7710, with an upper reference limit <34,2 pg/mL.

To estimate mortality risk, the GRACE score was calculated and said low risk if it was ≤108, intermediate risk if between 109 and 140, and high risk if >140.^13^ The definition of improvement was purely clinical characterized by the disappearance of clinical signs and especially the discharge of living patients from the hospital around the 14th day on average.

### 2.3 Statistical analysis

The data were collected from a pre‐established survey form, from the patient files. Then, they were exploited by the software Epi Info™ version 3.5.4 (United States Centers for Disease Control and Prevention in Atlanta, Georgia). The qualitative and quantitative variables are expressed, respectively, as a percentage and as an average with its standard deviation. We performed a statistical analysis comparing the different variables between with and without T2DM. The test was significant if the *p* value is less than .05.

## 3 RESULTS

During the study period, there were 3137 patients hospitalized in the Internal Medicine and Cardiovascular Diseases departments of Soavinandrina Hospital Center, for all reasons combined. One hundred and thirty patients were included in the study who met the eligibility criteria, giving an overall prevalence of NSTE‐ACS of 4.1%. Among them, 68 (52.3%) patients were with T2DM and 62 (47.7%) without T2DM.

Table 1 presents the general characteristics of the study population. The diabetic subjects were slightly younger than the nondiabetics (63.3 versus 64.5 years; *p* value = .0002). No significant gender difference was found between the two groups (*p* value = .2989). Hypertension was significantly more frequent in patients with T2DM than without T2DM (OR 2.92 [1.23–7.25]; *p* = .0041), as was overweight/obesity (4.39 [1. 72–12.4]; *p* = .0002). In 47 patients (69.1%), diabetes was already known with a mean duration of 7.6 ± 7.5 years (extremes: 1 year and 36 years). Mean Hb A1c was 8.8 ± 2.5% (range: 5.2 to 17.7%). Only 33.8% of patients had no associated complications.

**TABLE 1:** General characteristics of the population studied

Variables	With diabetes n1 = 68	Without diabetes n2 = 62	OR [95% CI]	*p* value
Male gender, *n* (%)	42 (61.8)	42 (67.7)	0.77 [0.34–1.68]	.2989
Mean age, years	63.3 ± 8.3	64.5 ± 11.4	‐‐‐	.0002^a^
Cardiovascular risk factors				
Hypertension, *n* (%)	56 (82.4)	38 (61.3)	2.92 [1.23–7.25]	.0041^a^
Smoking, *n* (%)	28 (41.2)	22 (35.5)	1.27 [0.59–2.75]	.3138
Dyslipidemia, *n* (%)	38 (55.9)	30 (48.4)	1.35 [0.63–2.85]	.2487
Microalbuminuria, *n* (%)	15 (22.1)	0 (0)	ND	<.0001^a^
Overweight/obesity, *n* (%)	27 (27.9)	8 (12.9)	4.39 [1.72–12.4]	.0002^a^
Type 2 diabetes mellitus				
Known, *n* (%)	47 (69.1)	‐‐‐	‐‐‐	‐‐‐
Mean duration, years	7.6 ± 7.5	‐‐‐	‐‐‐	‐‐‐
Mean Hb A1c, %	8.8 ± 2.5	‐‐‐	‐‐‐	‐‐‐
Hb A1c ≥7%, *n* (%)	49 (72.1)	‐‐‐	‐‐‐	‐‐‐
Associated degenerative complications				
None, *n* (%)	23 (33.8)	‐‐‐	‐‐‐	‐‐‐
Nephropathy, *n* (%)	31 (45.6)	‐‐‐	‐‐‐	‐‐‐
Neuropathy, *n* (%)	25 (36.8)	‐‐‐	‐‐‐	‐‐‐
Retinopathy, *n* (%)	20 (29.4)	‐‐‐	‐‐‐	‐‐‐
Arteriopathy of LL and/or SAT, *n* (%)	23 (33.8)	‐‐‐	‐‐‐	‐‐‐
Ischaemic stroke, *n* (%)	7 (10.3)	‐‐‐	‐‐‐	‐‐‐

Abbreviations: CI, confidence interval; Hb A1c, Glycated haemoglobin; LL, Lower limbs; OR, Odds ratio; SAT, Supra‐aortic trunk.

^a^ *p* value <.05.

Table 2 shows the clinical characteristics of non‐ST‐segment elevation acute coronary syndrome. Mean heart rate was slightly higher in diabetics than in non‐diabetics (*p* value = 0.0104). Chest discomfort was significantly atypical in patients with T2DM (OR 5.57 [2.21–15.7]; *p* value <.0001). Killip I was less frequent in patients with T2DM than without T2DM (OR 0.46 [0.21–1.01]; *p* = .0264).

**TABLE 2:** Clinical characteristics of non‐ST‐segment elevation acute coronary syndrome

Variables	With diabetes n1 = 68	Without diabetes n2 = 62	OR [95% CI]	*p* value
Vital parameters at admission				
Mean SBP, mmHg	132.4 ± 31	123 ± 21	‐‐‐	.0886
Mean DBP, mmHg	80.1 ± 16.5	77.5 ± 13	‐‐‐	.1608
Mean heart rate, bpm	87.5 ± 17	82.3 ± 17.3	‐‐‐	.0104^a^
Mean arterial oxygen saturation, %	94.4 ± 4.3	95.5 ± 2.3	‐‐‐	.3925
Clinical signs				
Chest discomfort, *n* (%)	43 (63.2)	42 (67.7)	0.82 [0.37–1.79]	.3617
Atypical chest discomfort, *n* (%)	31 (45.6)	8 (12.9)	5.57 [2.21–15.7]	<.0001^a^
Breathlessness, *n* (%)	36 (52.9)	26 (41.9)	1.55 [0.73–3.31]	.1402
Bilateral pulmonary crepitations, *n* (%)	30 (44.1)	16 (25.8)	2.25 [1.02–5.14]	.0224^a^
Gallop rhythm, *n* (%)	26 (38.2)	16 (25.8)	1.77 [0.79–4.06]	.0921
Ankle swelling, *n* (%)	24 (35.3)	18 (29.0)	1.33 [0.59–3.00]	.2831
Hepatojugular reflux, *n* (%)	24 (35.3)	14 (22.6)	1.86 [0.81–4.41]	.0805
Elevated jugular venous pressure, *n* (%)	19 (27.9)	12 (19.4)	1.61 [0.66–4.05]	.1733
Classification de Killip				
I, *n* (%)	36 (52.9)	44 (71.0)	0.46 [0.21–1.01]	.0264^a^
II, *n* (%)	23 (33.8)	16 (25.8)	0.46 [0.64–3.38]	.2107
III, *n* (%)	3 (4.4)	0 (0)	ND	.1401
IV, *n* (%)	6 (8.8)	2 (3.3)	2.88 [0.49–30.3]	.1689

Abbreviations: bpm, beat per minute; CI, Confidence Interval; DBP, Diastolic blood pressure; ND, not defined; OR, Odds ratio; SBP, systolic blood pressure.

^a^ *p* value <.05.

Table 3 presents the paraclinical characteristics of non‐ST‐segment elevation acute coronary syndrome. Diabetes exposed much more to extended anterior damage (OR 2.11 [1.02–4.98]; *p* = .0402), septal (OR 3.64 [1.41–10.3]; *p* = .0015) and ≥2 leads ((OR 2.41 [1.02–5.92]; *p* = .0219) on the ECG; a more marked increase in cardiac troponin (*p* < .0001) and an increase in left ventricular filling pressure (LVFP) (OR 3.39 [1.51–7.90]; *p* = .0006) on transthoracic echocardiography and a higher mean GRACE score (*p* = .0016).

**TABLE 3:** Paraclinical characteristics of non‐ST‐segment elevation acute coronary syndrome

Variables	With diabetes n1 = 68	Without diabetes n2 = 62	OR [95% CI]	*p* value
Leads affected on ECG				
Inferior, *n* (%)	29 (42.6)	28 (45.2)	0.90 [0.43–1.91]	.4554
Extended anterior, *n* (%)	26 (38.2)	14 (22.6)	2.11 [1.02–4.98]	.0402^a^
Septal, *n* (%)	24 (35.3)	8 (12.9)	3.64 [1.41–10.3]	.0015^a^
Anterior, *n* (%)	9 (13.2)	14 (22.6)	0.52 [0.18–1.43]	.1221
Apical, *n* (%)	5 (7.4)	6 (9.7)	0.74 [0.16–3.09]	.4352
Posterior, *n* (%)	3 (4.4)	8 (12.9)	0.31 [0.05–1.39]	.0768
≥2 leads affected	25 (36.8)	12 (19.4)	2.41 [1.02–5.92]	.0219^a^
Cardiac troponin				
Mean at 0 h, pg/ml	10779.6 ± 26139.5	2466.7 ± 6134.3	‐‐‐	<.0001^a^
Echocardiography results				
Segmental hypokinesia, *n* (%)	44 (64.7)	44 (71.0)	0.75 [0.33–1.67]	.2830
Global hypokinesia, *n* (%)	17 (25.0)	8 (12.9)	2.23 [0.82–6.53]	.0627
LVEF preserved, *n* (%)	29 (42.6)	36 (58.1)	0.53 [0.25–1.14]	.0568
LVEF reduced, *n* (%)	25 (36.8)	22 (35.5)	1.05 [0.48–2.31]	.5126
LVEF mildly reduced, *n* (%)	13 (19.1)	6 (9.7)	2.19 [0.71–7.56]	.1007
High LVFP, *n* (%)	34 (50.0)	14 (22.6)	3.39 [1.51–7.90]	.0006^a^
Mean GRACE score	134.4 ± 33.9	121.8 ± 35	‐‐‐	.0016^a^

Abbreviations: CI, Confidence Interval; ECG, electrocardiogram; GRACE, Global Registry of Acute Coronary Events; LVEF, left ventricular ejection fraction; LVFP, left ventricular filling pressure; OR, Odds ratio.

^a^ *p* value <.05.

Table 4 shows the drug treatment received during hospitalization for non‐ST‐segment elevation acute coronary syndrome and the evolution. There was no significant difference in medical treatment during hospitalization between the two groups. During hospitalization, 77.9% of patients with type 2 diabetes had followed lifestyle and dietary measures with insulin therapy and 22.1% lifestyle and dietary measures alone. All patients had received antithrombotic treatment combining clopidogrel (loading dose of 300 mg orally, followed by a maintenance dose, once daily) and aspirin (100 mg orally, once daily). In‐hospital mortality rates for diabetics and nondiabetics were 7.4% and 3.2%, respectively. However, the statistical test was not significant (*p* = .2600).

**TABLE 4:** Drug treatment during hospitalization for non‐ST‐segment elevation acute coronary syndrome and evolution

Variables	With diabetes (n1 = 68)	Without diabetes (n2 = 62)	OR [95% CI]	*p* value
Drug treatment				
ACE inhibitors, *n* (%)	41 (60.3)	42 (67.7)	0.72 [0.32–1.57]	.2421
Angiotensin receptor blocker, *n* (%)	26 (38.2)	18 (29.0)	1.51 [0.68–3.38]	.1783
Diuretics, *n* (%)	41 (60.3)	32 (51.6)	1.41 [0.67–3.02]	.2063
Beta‐blockers, *n* (%)	50 (73.5)	48 (77.4)	0.81 [0.33–1.95]	.3788
Spironolactone, *n* (%)	19 (27.9)	14 (22.6)	1.33 [0.56–3.21]	.3092
Antiplatelet agents, *n* (%)	68 (100)	62 (100)	‐‐‐	‐‐‐
Statins, *n* (%)	61 (89.7)	58 (93.5)	0.60 [0.12–2.52]	.3211
Anticoagulation by LMWH, *n* (%)	55 (80.9)	54 (87.1)	0.61 [0.21–1.79]	.4696
Outcome				
Mean duration of hospitalization, days	13.3 ± 6.6	13.6 ± 6.5	‐‐‐	.1955
Released with improvement, *n* (%)	61 (89.7)	58 (93.6)	0.61 [0.12–2.52]	.3211
Released against medical advice, *n* (%)	2 (2.9)	2 (3.2)	0.91 [0.06–12.9]	.6547
Deceased in hospital, *n* (%)	5 (7.4)	2 (3.2)	2.36 [0.37–25.7]	.2600

Abbreviations: ACE, Angiotensin‐converting enzyme; CI, Confidence Interval; LMWH, Low‐molecular‐weight heparin; OR, Odds ratio.

^a^ *p* value <.05.

## 4 DISCUSSION

The overall prevalence of NSTE‐ACS in our patients was 4.1%, 52.3% of whom had T2DM. Among African studies, in Ivory Coast, it was 3.8% and only 26.4% of them had diabetes mellitus.^3^ In Tunisia, 47.2% of patients hospitalized for NSTE‐ACS were diabetic.^14^ In Western countries, 20%–30% of subjects suffering from ACS have diabetes mellitus, which increases considerably during non‐ST‐segment elevation myocardial infarction.^15^, ^16^, ^17^ This disparity in the overall prevalence of ACS and the proportion of diabetes mellitus could be explained by the difference in the population studied but above all in the criteria for making the diagnoses.

The male predominance remains classic in this study as in other African studies.^2^, ^18^, ^19^ However, the gender distribution was comparable between the two groups of our patients. This was not the case in a Tunisian study in which the proportion of women was higher among diabetics than nondiabetics (44.9% versus 31.1%; *p* value = .024).^14^ Indeed, the risk associated with coronary artery disease is higher in diabetic women than in men, in view of the cancellation of protection before menopause in terms of coronary artery disease and the risk of stroke in women diabetic.^20^ In our study, NSTE‐ACS occurred significantly at a younger age in diabetics than in nondiabetics. In addition, middle‐aged diabetics are two‐ to fourfold more likely to develop coronary artery disease than nondiabetics. Being diabetic confers a risk of ACS comparable to that of a non‐diabetic subject 10 years older.^21^, ^22^ This would support systematic screening for coronary artery disease in diabetics regardless of their age.

In our study as in the literature,^14^, ^23^ hypertension was significantly more frequent in diabetics than in nondiabetics. Indeed, insulin resistance and reactive hyperinsulinaemia, characteristic of T2DM, contribute to the appearance of hypertension by inappropriate activation of the renin‐angiotensin‐aldosterone system and sympathetic nervous system, mitochondria dysfunction, excessive oxidative stress and systemic inflammation.^24^ Other authors had objectified a high prevalence of dyslipidemia in diabetics than in nondiabetics with *p* value <.001.^14^ Since insulin resistance and relative insulin deficiency appear to play an important role in the pathophysiology of lipid abnormalities observed in T2DM. And insulin performs essential functions in the control of lipid metabolism. And chronic hyperglycemia also seems to be involved.^25^ In addition, cigarette smoking impacts body weight and composition, peripheral insulin sensitivity, and pancreatic β cell function, thus increasing the risk of developing T2DM.^26^ The prevalence of overweight/obesity and microalbuminuria was significantly higher in our diabetics than in non‐diabetics. This was consistent with the findings of the other series.^14^, ^23^ Indeed, obesity is both a factor of insulin resistance at the origin of the occurrence of T2DM and an independent cardiovascular risk factor, thus contributing to the occurrence of an ACS.^27^ In short, our diabetics accumulated other cardiovascular risk factors, thus requiring early and adequate management.

The mean duration of diabetes in our patients was significantly lower than that of the British study.^28^ This could be linked to the frequent delay in diagnosing diabetes in low‐income countries like ours and to the ageing of the population in developed countries. In addition, the glycemic control of our patients was poor, favouring the occurrence of these degenerative complications, especially microvascular. However, diabetes mellitus and hyperglycemia on admission increases the risk of both short‐ and long‐term mortality of patients with ACS, whether the patient has diabetes or not.^29^, ^30^ Thus, early and adequate management of the diabetic disease remains essential.

In our study as in the literature,^31^ on admission, patients with T2DM had a significantly higher heart rate than without T2DM. Indeed, cardiovascular autonomic neuropathy, a very common complication of diabetes (20%–65% by age and diabetes duration), can manifest as permanent tachycardia,^32^ and thus contribute to this elevation in heart rate compared to nondiabetics. The clinic of ACS during T2DM is characterized by atypia of chest discomfort in our patients as in those of other studies.^33^ Patients with T2DM were significantly more prone to pulmonary crepitation. According to hemodynamic status, the Killip classification was less severe in nondiabetics than in diabetics. Indeed, diabetics with ACS are at higher risk of developing heart failure and cardiogenic shock.^34^ Thus, one must always be wary of the atypical clinical presentation of sudden and severe onset in diabetics.

In our diabetic patients, the leads significantly most affected on the ECG were extended anterior, septal and with at least two leads. This could be explained by the often‐multifocal coronary involvement in diabetes mellitus.^35^ The increase in cardiac troponin is significantly more marked in our diabetics than in nondiabetics. Indeed, cardiac troponin elevations above the 99th percentile measured by a highly sensitive test have been frequently encountered in a T2DM patient population. Their levels appeared stable over time.^36^ Transthoracic echocardiography allowed us to identify abnormalities indicative of ischaemia or myocardial necrosis. Only the LVFP was statistically higher in our diabetics. However, elevated glycated haemoglobin is associated with reduced left ventricular ejection fraction. And chronic hyperglycaemia appears to have a stronger association with cardiovascular mortality and heart failure, linked to an increased risk of recurrent ischaemic events.^37^

Mean GRACE score was significantly higher in our diabetics than nondiabetics. Indeed, the increase in heart rate, serum creatinine level and initial cardiac troponin are components of the GRACE score and significantly more frequent in patients with type 2 diabetes13. The non‐invasive pharmacological management and the short‐term outcome were comparable between the two groups of our study population. However, diabetes is an independent factor in increased short‐term and long‐term mortality and recurrence in patients with ACS.^34^, ^38^

### 4.1 Limitations of the study

Our study is limited by its monocentric nature and the size of the unrepresentative sample, which is a source of recruitment bias and does not allow for more in‐depth statistical analysis. Its retrospective nature also does not make it possible to assess certain important information that did not appear in the medical records, such as the performance of the coronary angiography and the angioplasty. This is linked to the inadequacy of our technical platforms and the inaccessibility to these interventional techniques.

## 5 CONCLUSION

To conclude, NSTE‐ACS was frequent in patients with younger T2DM, accumulating more cardiovascular risk factors, with more accelerated heart rate, atypical chest discomfort, pulmonary crepitations, and higher GRACE score. The electrical coronary involvement was multifocal, with a significantly higher increase in cardiac troponin and an increase in LVFP on echocardiography.

In primary prevention, the adequate management of all cardiovascular risk factors, including diabetes mellitus, thus makes it possible to reduce the occurrence of this disease. In secondary prevention, it must be multidisciplinary involving cardiologists, endocrinologists, resuscitators and general practitioners. Given the limitations of the study, another study focussed on performing invasive treatment such as coronary angiography, thrombolysis and angioplasty, will be necessary in order to know other lesional particularities in Malagasy diabetics. The population follow‐up of the present study is also interesting to determine the long‐term outcome without interventional management.

## AUTHOR CONTRIBUTIONS

**Sitraka Angelo Raharinavalona:** Conceptualization (equal); data curation (equal); formal analysis (equal); investigation (equal); methodology (equal); software (equal); supervision (equal); validation (equal); writing – original draft (equal); writing – review and editing (equal). **Rija Eric Raherison:** Validation (equal); writing – review and editing (equal). **Thierry Razanamparany:** Software (equal); visualization (equal). **Tsikinirina Valisoa Randrianomanana:** Investigation (equal); software (equal). **Andrianirina Dave Patrick Rakotomalala:** Validation (equal); writing – review and editing (equal).

## FUNDING INFORMATION

This study was not supported by any funding source.

## CONFLICT OF INTEREST

The authors have declared that they have no competing interest.

## Data Availability

The data sets used and/or analysed during the current study are available from the corresponding author on reasonable request.
